# Future U.S. supply of Mo-99 production through fission based LEU/LEU technology

**DOI:** 10.1007/s10967-015-4090-9

**Published:** 2015-04-01

**Authors:** James Welsh, Carmen I. Bigles, Alejandro Valderrabano

**Affiliations:** Coquí RadioPharmaceuticals Corp., 1172 South Dixie Hwy., Suite 335, Coral Gables, FL 33146 USA

**Keywords:** U.S. supply of Mo-99, Molybdenum-99, Nuclear medicine, Production through fission based LEU/LEU, Technetium-99, Diagnosis

## Abstract

Coquí RadioPharmaceuticals Corp. (Coquí) has the goal of establishing a medical isotope production facility for securing a continuous domestic supply of the radioisotope molybdenum-99 for U.S. citizens. Coquí will use an LEU/LEU proven and implemented open pool, light-water, 10 MW, reactor design. The facility is being designed with twin reactors for reliability an on-site hot lab chemical processing and a waste conditioning area and a possible generator producing radio-chemistry lab. Coquí identified a 25 acre site adjacent to an existing industrial park in northern central Florida. This land was gifted and transferred to Coquí by the University of Florida Foundation. We are in the process of developing licensing documents related to the facility. The construction permit application for submission to the U.S. Nuclear Regulatory Commission is currently being prepared. Submission is scheduled for mid to late 2015. Community reaction to the proposed development has been positive. We expect to create 220 permanent jobs and we have an anticipated to be operational by 2020.

## Introduction

Molybdenum-99 (Mo-99) and its decay product, technetium-99m (Tc-99), the most widely used medical radioisotope in the world, are used in life saving medical diagnostic imaging techniques that enable precise and accurate early detection and management of diseases such as heart conditions and cancer, all in a non-invasive manner. Tc-99 medical imaging techniques account for over 80 % of all nuclear medicine procedures, representing over 30 million worldwide and 14 million domestic procedures every year [1–4]. Primary uses include detection of heart disease, cancer, study of organ structure and function, among other applications [4]. These imaging techniques can significantly impact medical decisions by providing predictive information about the likely success of alternative therapy options or whether there is a need for surgical intervention. This nuclear isomer is also used in research for advancements in medicine. Furthermore, alternatives to Tc-99 are very costly and may result in a higher dose rate to the patient [1].

The United States has limited domestic production source of Mo-99, and therefore it depends on foreign imports of Mo-99. Without its own production source, the U.S. is subject to increased risk of supply chain disruptions, which could create a potential crises where U.S. citizens could face delays is diagnosis and treatment. The U.S. is also confronted with the security risks of exporting highly enriched uranium (HEU), for humanitarian purposes which is historically used in production of Mo-99 but is weapons grade uranium and thus raises some proliferation concerns in the case that it is derailed from its intended destination. Through the Global Threat Reduction Initiative, the U.S. is promoting the use of low enriched uranium (LEU) for Mo-99 production [4].

The American Medical Isotopes Production Act of 2012 (AMIPA) was approved as part of the 2013 National Defense Authorization Act for Fiscal Year 2013 (H. R. 4310). AMIPA provides that the Secretary of the United States Department of Energy (DOE) shall carry out a technology neutral program to evaluate and support private sector projects for the production within the United States of significant quantities of Mo-99 for medical use without the use of HEU [4].

As part of the program and pursuant to AMIPA, the Secretary of DOE provides assistance for the development of fuels, targets, and processes, for domestic Mo-99 production methods that do not use HEU. Moreover, the Secretary may establish a uranium lease and take-back program to make LEU available through lease contracts for irradiation for the production of Mo-99 for medical use (commonly referred to as the “Lease and Take-Back” program) [4]. Although the government is providing marginal support it is clear that to ensure a future reliable supply of Mo-99 the industry must start its long overdue transition to the private sector.

Our company, Coquí RadioPharmaceuticals Corporation (Coquí), has the specific goal of establishing a dedicated medical isotope production facility (MIPF) in order to secure a significant and continuous domestic supply of the radioisotope Mo-99 for patients. The company will use LEU/LEU technology and irradiation techniques that have been successfully demonstrated, proven and implemented in several countries around the world and whose end products have obtained the approval of the Food and Drug Administration (FDA).

## Coquí’s proven technology

Coquí will be developing and operating the first domestically, dedicated Mo-99 and radioisotope pharmaceutical production facility utilizing proven technology that relies on LEU thus:

- Providing a continuous source of Mo-99 for the world’s nuclear medicine industry;
- Advancing nuclear medicine by establishing a state of the art production facility;
- Contributing to national security by reducing the need for exports of HEU for Molybdenum production; and,
- Bringing needed employment, new sources of industry and economic development.

The techniques employed by the company have a focus on reliably and optimization for commercial scale production.

Coquí will couple existing proven technology with tested designs to build its facility and will have the capability of supplying the U.S. market needs of Mo-99. The MIPF consists of twin, small nuclear reactors that operate at less than 10 MW, a radioisotope processing plant, and a waste conditioning plant. LEU targets will be fissioned in the reactors, and then the already fissioned targets will be taken to the processing cells where Mo-99 will be extracted. Once the Mo-99 is extracted, it will be in the form of an aqueous solution of sodium molybdate.

## Site description and progress

**Fig. 1:**
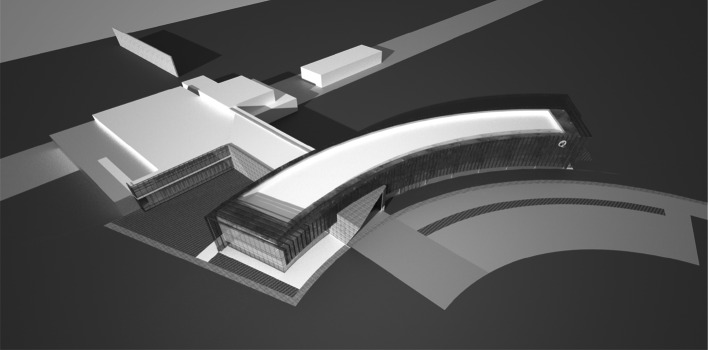
This is a rendering of the footprint of the facility

**Fig. 2:**
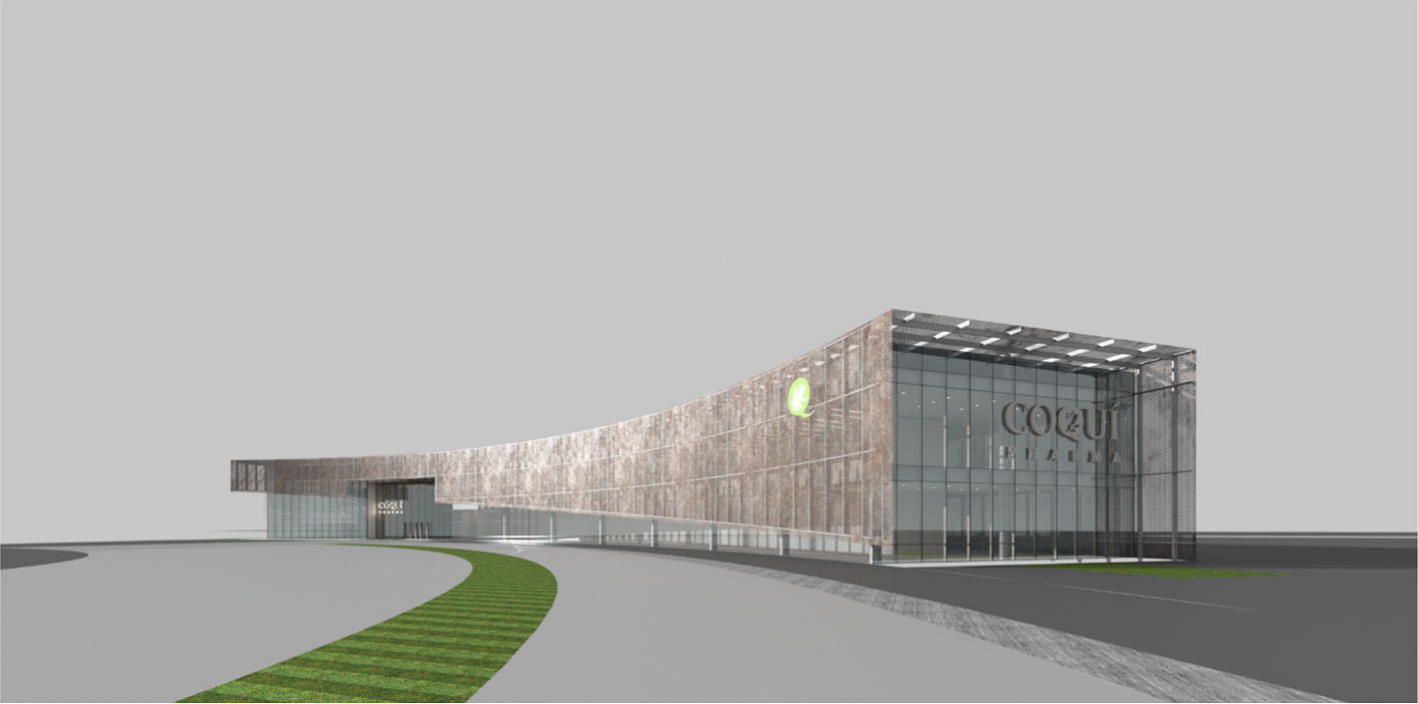
Conceptual design of the 100,000 square feet Coquí Medical Isotope Production Facility

The approximately 100,000 square foot MIPF (see Figs. 1, 2) will be situated approximately 2 miles southeast of the center of the City of Alachua in Alachua County, Florida. It consists of an approximately 25-acre parcel in Progress Corporate Park, which was transferred to Coquí from the University of Florida Foundation. Surrounding property consists of vacant agricultural land immediately to the west, agricultural and low-density residential land to the south, the San Felasco Hammock Preserve State Park immediately to the east, and office buildings, along with manufacturing and R&D operations, to the north in Progress Corporate Park. The City of Gainesville, approximately 10 miles to the southeast, is the only major population center with greater than 25,000 people located within Alachua County. The City of Alachua and the City of High Springs, approximately 10 miles to the northwest, are the only other population centers with more than 5000 people.

Construction of the facility will be primarily concrete and steel. The building will be attached to local electricity, water, and sewer utilities, and will house redundant uninterrupted power generators to provide backup power to all reactor control systems for unplanned outages. The building is designed to meet all local and federal construction codes. Demonstration that the facility design and characteristics of the Site meet applicable safety requirements will be made in the Preliminary Safety Analysis Report (PSAR). Baseline environmental conditions and potential impacts of construction, operations and decommissioning of the MIPF are detailed in the Environmental Report (ER) which will be submitted approximately 2–3 months before the PSAR submission.

Research concerning the regional context of the site has been performed. This includes analysis of socioeconomic and demographic data, climate and weather, regional geology and hydrogeology, and ecological data. Local and site-specific data are being collected. Methods utilized during both research and data collections are based upon technical guidance, professional standards of care and quality program constraints.

Site characterization fieldworks, to support submission of both the PSAR and ER, have concluded. Investigation has found no surface water or wetlands on the property. Archeological fieldwork and research has resulted in no significant findings associated with the site. Outreach with Tribal governments has been performed. Geophysical surveys, geotechnical borings and soil analysis have been completed. Ecological and groundwater monitoring where concluded at the end of 2014.

## Community outreach and impact

Proactive coordination regarding the site with the Gainesville Area Chamber of Commerce, Council for Economic Outreach, University of Florida, Alachua County, and the City of Alachua is ongoing. Outreach to additional stakeholders, such as neighborhood meetings and a speaker’s bureau, have begun and will keep on taking place over the next year. Community reaction has been positive to date.

The economic impact of the construction phase of the project is anticipated to generate, in a non-recurring nature, approximately 1824 jobs, pay about $82.5 million in labor income into the local economy, create local total value added of about $113 million, resulting into a total output of $246 million into the local economy. The tax revenue impact is expected to be about $6 million to the state of Florida and $1.5 million to Alachua County over the construction and installation phase of the project.

The ongoing employment impact of the facility will be approximately 220 jobs added to the local economy. This will result in an approximate labor income of $14.4 million annually, total local value added of $17.6 million annually, and a total output of $31.2 million annually. Annual operations are expected to generate about $0.78 million in sales and use tax revenue to Florida and as much as $0.2 million to Alachua County.

## Licensing

Coquí is in the pre-application stage with the NRC. It’s most recent public meeting with the NRC was in October 2014, where Coquí provided an update on the project. Coquí is preparing licensing documents and submittals in conformance with NRC regulations and guidance and continues to keep the NRC informed on milestones of progress associated with license submittal. It is anticipated that the entire Construction Permit application will be submitted in mid to late 2015. The Construction Permit application review period is expected to take 18–24 months. Coquí will then need to submit an application to the NRC for an Operating License. Communication with the NRC is active, and frequent. Our next meeting is scheduled for March 26, 2015.

## Closing statements

**Fig. 3:**
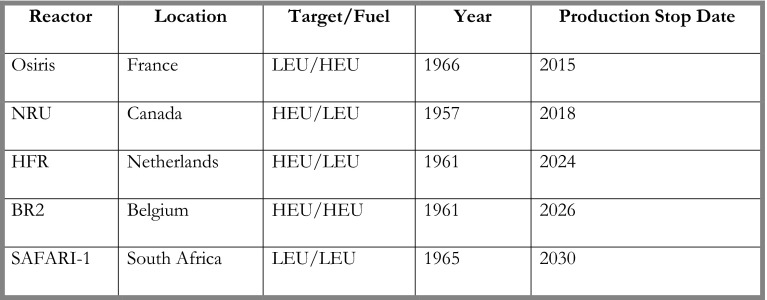
Stop date as per OECD Publication “Medical Isotope Supply in the Future: Production Capacity and Demand Forecast for the Mo-99/Tc-^99m^ Market, 2015–2020” (April 2014). Canada listed as 2016 under OECD but the Canadian government recently announced 2018

The driving philosophy of Coquí is to assist in saving lives while operating a sustainable business model. Distributors of Tc-99 have been forced to ration supplies among and within countries, leaving hundreds of thousands of people without access to crucial diagnostic procedures. With the anticipated closure of additional Mo-99 production reactors, see Fig. 3, the pressures for a continuous supply of Mo-99 and Tc-99 will increase.

Moreover, several countries have signed the Joint Declaration on the Security of Supply of Medical Radioisotopes highlighting that the production of Mo-99 and its daughter product Tc-99m depends largely on a small number of reactors that are ageing and facing unplanned outages, planned refurbishment outages or planned permanent shutdowns, which increases the risk of disruption of the supply chain, unless new infrastructure is developed to replace these facilities before they shut down [5].

With such a delicate supply chain and no domestic production source, the U.S. is highly vulnerable to supply disruptions, which could impact thousands of people in the U.S. daily. Therefore it is critical that a reliable production source of Mo-99 be established, using proven and reliable technology. Coquí is acting quickly to implement this reliable, secure and continuous flow of a domestic significant supply of Mo-99 for the U.S. patient.

We are currently preparing the submission of our Environmental Report and Preliminary Safety Analysis Report to the U.S. Nuclear Regulatory Commission. Twin reactors, radioisotope processing plant and waste management area. We are on schedule for a mid to late 2015 submission. We are also preparing the construction documents for submittal to the local and state regulatory bodies. As well as all agencies that affects our operations. In regards to the operation license application the specific date will be determined as project progresses.
